# Intergenerational nutrition benefits of India’s national school feeding program

**DOI:** 10.1038/s41467-021-24433-w

**Published:** 2021-07-12

**Authors:** Suman Chakrabarti, Samuel P. Scott, Harold Alderman, Purnima Menon, Daniel O. Gilligan

**Affiliations:** Poverty Health and Nutrition Division, International Food Policy Research Institute, Washington, DC USA

**Keywords:** Developing world, Economics

## Abstract

India has the world’s highest number of undernourished children and the largest school feeding program, the Mid-Day Meal (MDM) scheme. As school feeding programs target children outside the highest-return “first 1000-days” window, they have not been included in the global agenda to address stunting. School meals benefit education and nutrition in participants, but no studies have examined whether benefits carry over to their children. Using nationally representative data on mothers and their children spanning 1993 to 2016, we assess whether MDM supports intergenerational improvements in child linear growth. Here we report that height-for-age z-score (HAZ) among children born to mothers with full MDM exposure was greater (+0.40 SD) than that in children born to non-exposed mothers. Associations were stronger in low socioeconomic strata and likely work through women’s education, fertility, and health service utilization. MDM was associated with 13–32% of the HAZ improvement in India from 2006 to 2016.

## Abstract (web summary)

India’s national school feeding program is the largest of its kind in the world, but the long-term program benefits on nutrition are unknown. Here, the authors show intergenerational program benefits, in that women who received free meals in primary school have children with improved linear growth.

## Introduction

Globally, 149 million children are too short for their age and over half of these children live in Asia^1^. Within India, 38% of children were stunted in 2015–2016 (ref. ^2^). Linear growth failure is a marker of chronic undernutrition and multiple pathological changes which, together, have been termed the ‘stunting syndrome’^3^. Stunted children are at risk of not reaching their developmental potential, thus stunting has large implications for human capital and the economic productivity of entire societies^4–6^. The World Health Assembly set the ambitious target of reducing childhood stunting by 40% from 2010 to 2025 (ref. ^7^), a target that likely will not be met^4^. Thus, it is imperative to understand how countries can accelerate progress toward stunting reduction.

Though much focus has been placed on nutrition-specific interventions during the 1000-day period from conception to the child’s second birthday, investments across multiple life periods and which address underlying determinants are also important to achieve stunting reductions^8,9^. Interventions may work directly through maternal–child biological pathways or indirectly through socioeconomic mechanisms. In India, women’s height and educational attainment are among the strongest predictors of child stunting^10–15^.

In the Indian context, a candidate intervention which potentially improves both women’s height and education—and which, therefore, may lead to reductions in stunting among children born to these women—is the national school feeding program, the Mid-Day Meal (MDM) scheme^16^. Launched in 1995 by the Government of India, the MDM scheme provides a free cooked meal to children in government and government-assisted primary schools (classes I–V; ages 6–10 years). The mandated minimum meal energy content is 450 kcal and the meal must contain 12 g of protein. In 2016–2017, 97.8 million children received a free cooked meal through the scheme every day, making the MDM scheme the largest school feeding program in the world^17^.

Econometric evaluations of India’s MDM scheme have shown a positive association with beneficiaries’ school attendance^18,19^, learning achievement^20^, hunger and protein-energy malnutrition^21^, and resilience to health shocks such as drought^22^—all of which may have carryover benefits to children born to mothers who participated in the program. We are not aware of studies that have explored whether program benefits for the MDM or similar programs in other countries extend to the next generation. Filling this research gap is critical, as (1) stunting carries over from one generation to the next and is therefore optimally studied on a multigenerational time horizon^23–26^, (2) school feeding programs are implemented in almost every country^27^, and (3) social safety nets such as India’s MDM scheme have the potential for population-level stunting reduction as they are implemented at scale and target multiple underlying determinants in vulnerable groups^28^.

At a broader level, a substantial literature documents effects of cash transfer programs on education of girls in low- and lower-middle-income countries^29^. While transfer programs clearly address food security, their track record on improving anthropometry is mixed at best, possibly because evaluations focus on relatively short-term impacts^28,30^. However, even in the United States, a timely transfer—for example, the Supplemental Nutrition Assistance Program—has been shown to have health benefits over time^31^. Other studies document effects of cash transfers, health insurance, and other programs for children in beneficiary households on future adult outcomes such as incomes, achieved schooling^32^, nutritional status^33,34^, and mortality^35^.

The described literature suggests a potential pathway through which school feeding programs and other cash transfer or in-kind safety nets focused on education may have intergenerational effects on child nutrition outcomes. Current frameworks for understanding the intergenerational transmission of health disparities advocate for a multi-generation approach that addresses parental socioeconomic status (SES), child and adolescent health and development, and young adult’s capacity for planning and future parenting^36^. However, since interventions to improve maternal height and education must be implemented years before those girls and young women become mothers, empirical assessment of the effectiveness of such programs for reducing undernutrition among future offspring is challenging.

This paper studies the intergenerational nutrition benefits of India’s MDM scheme. We use seven population level datasets spanning 1993 to 2016, including multiple rounds of National Sample Surveys of Consumer Expenditure (NSS-CES), National Family Household Surveys (NFHS), and India Human Development Surveys (IHDS). We match cohorts of mothers by state, birth year, and SES with data on MDM coverage measured as the proportion of primary-school-age girls receiving MDM using data from the NSS-CES. Birth cohort fixed effects and controlled interrupted time series models are used to estimate the association of mother’s exposure to the MDM scheme with the nutritional status of her future children. We find that maternal cohorts living in areas with higher coverage of the MDM scheme are less likely to have stunted children than cohorts living in low coverage areas. This effect is robust to the inclusion of a broad set of controls at multiple levels and fixed effects. Controlled interrupted time series models confirm that the 14 states which rolled out MDM in the late-1990s experienced improvements in child height earlier than the rest of the nation, which scaled up MDM in the 2000s after the Supreme Court mandate. Plausibility is supported by our findings of MDM association with participants’ education, age at birth, number of children, use of antenatal care, and delivery in a medical facility.

## Results

### Program description and motivation

**Fig. 1:**
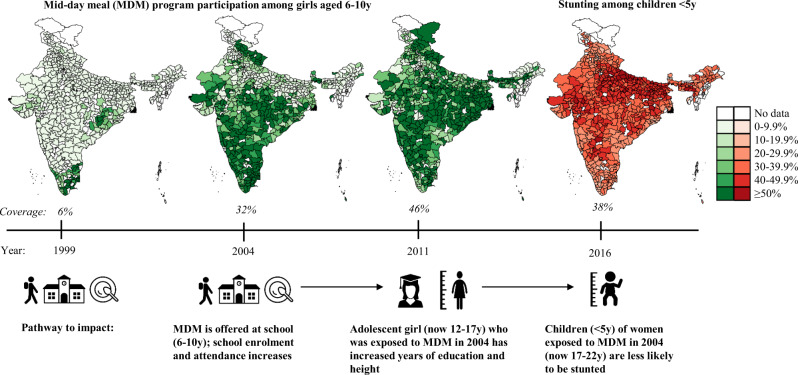
Overview of study design and proposed pathway. Coverage refers to the proportion of girls aged 6–10 years who received a MDM in school. Source for MDM program coverage data (green maps): NSS-CES 55 (2000), 61 (2005) and 68 (2012). Source for child stunting data (red map): NFHS4 (2016). MDM, mid-day meal. Source data are provided as a Source Data file.

The MDM scheme, initiated by the central government in 1995, was intended to cover all government schools under the National Programme of Nutritional Support for Primary Education^21^. Due to institutional challenges, only a few states scaled up the program immediately. NSS-CES data from 1999 show that only 6% of all girls aged 6–10 years received mid-day meals in school (Fig. 1). Between 1999 and 2004, program coverage increased in many states, largely due to an order from the Supreme Court of India directing state governments to provide cooked mid-day meals in primary schools^37^. In 2004, 32% of Indian girls aged 6–10 years were covered by the program. Finally, following a substantial increase in the budget allocation for the program in 2006, by 2011, 46% of girls aged 6–10 years benefited from the program. Coverage among boys was similar throughout this period. NSS-CES data show that substantial state variability in MDM rollout existed even ten years after the central mandate. A complete listing of state heterogeneity in program roll-out can be found in Supplementary Table 1.

**Fig. 2:**
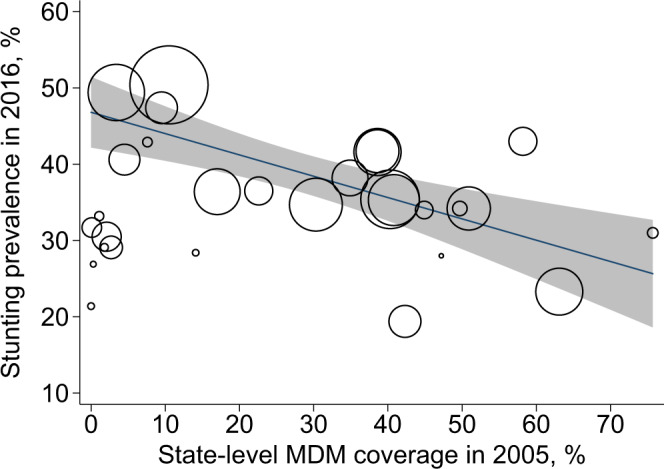
Association between stunting prevalence among children under 5 years old in 2016 and MDM coverage among girls 6–10 years old in 2005. Each circle represents an individual state in India, with the size representing the state population size. Fit line and shaded 95% confidence interval are also weighted by state population size. Sources: NFHS 4 (2016) for stunting data and NSS-CES 61 (2005) for MDM coverage data. MDM mid-day meal. Source data are provided as a Source Data file.

Our empirical exploration of the intergenerational benefits of the MDM scheme was motivated by the observation that stunting prevalence was lower among children aged 0–5 years in 2016 in states where MDM coverage was higher in 2005 (Fig. 2). The ability of historical MDM coverage to predict the prevalence of stunting in 2016 suggests that a mother’s exposure to the program during primary school may have future returns for her children. However, the observed association may be biased because policy variables in observational data are unlikely to be independent of latent individual and institutional characteristics^38^.

### Birth cohort fixed effects analyses

**Fig. 3:**
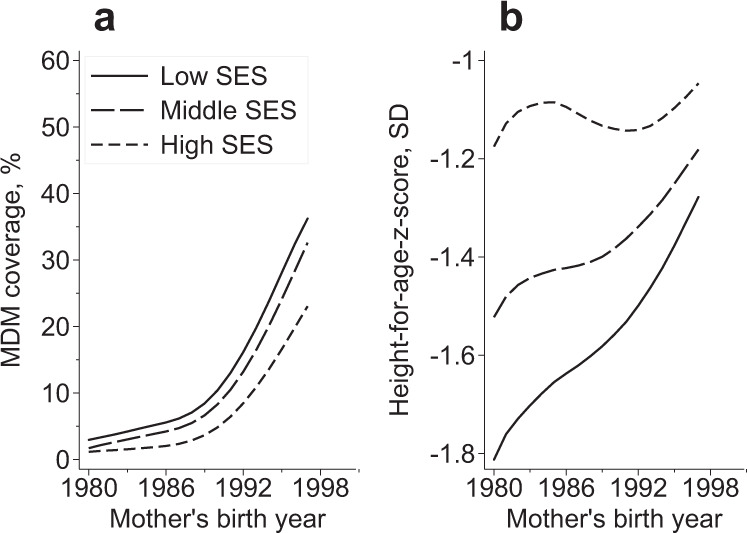
MDM coverage and child HAZ by mother’s birth year and socioeconomic status. Bottom 3 deciles are the poorest households in the sample and top 4 deciles are non-poor. MDM exposure of women born between 1980 and 1998 (**a**) and HAZ of children under 5 years old in 2016 of mothers born between 1980 and 1998 (**b**). Source of MDM coverage data: NSS-CES 50 (1994), 55 (2000), and 61 (2005). Source of HAZ data: NFHS 4 (2016). HAZ height-for-age *z*-score, MDM mid-day meal. Source data are provided as a Source Data file.

To inform the birth cohort fixed effects analysis, we examined coverage and scale-up of the MDM scheme and HAZ of children by mother’s birth year and SES. The rate of MDM scale-up across SES deciles moved in tandem with child HAZ along the mother’s birth year axis (Fig. 3). Later-born mothers from poor households were more likely to be exposed to the program than either earlier-born mothers or mothers from non-poor households (Fig. 3a). HAZ in children also increased with later mother’s birth year and was higher in non-poor households compared to poor households (Fig. 3b). The observed trends provide motivation for using MDM rollout by mother’s birth year as a source of variation that is time varying and cohort specific^39^.

**Fig. 4:**
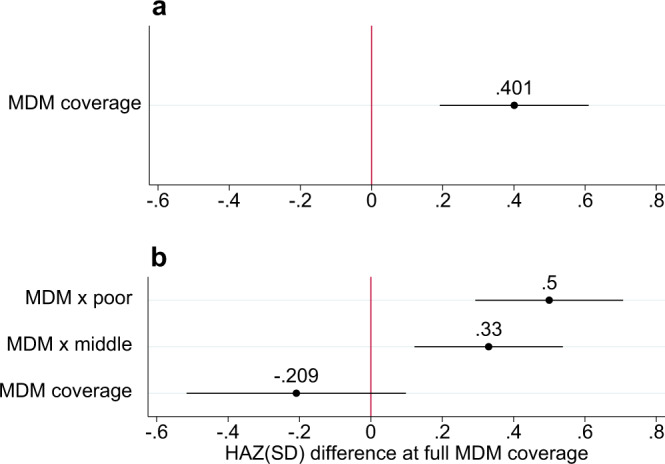
Relationship between MDM coverage and future child HAZ: birth cohort fixed effects model. Panel **a** shows the relationship between MDM coverage and future child HAZ in the birth cohort model (Eq. 1) while panel **b** shows the relative association across wealth strata (Eq. 2). The circles represent the point estimates and whiskers are 95% confidence intervals. Point estimates are interpreted as the difference in HAZ due to 100% exposure to the MDM scheme during primary school years for the relevant sample. Point estimates in panel **b** for MDM × poor and MDM × middle are the relative effect of 100% MDM coverage for that SES stratum compared to the average effect of 100% MDM coverage for the wealthiest four deciles (MDM coverage). MDM coverage is the proportion of girls born between 1980 and 1998, within state-specific socioeconomic status deciles, who reported receiving at least 10 meals free of cost at school in the previous month. All models control for child age, sex, birth order, maternal antenatal care (4+ visits), institutional birth, residence (urban/rural), religion, caste, access to services from the Integrated Child Development Services (dummies for receiving take home rations, child health check-ups, pre-school education, weight measurements, and nutrition counseling) and the Public Distribution System (household has a Below Poverty Line card to obtain subsidized food). The models include fixed effects for mother’s birth year, state, household wealth, and for state × mother’s birth year. All models cluster standard error estimates at the district level. Sources: NFHS 4 (2016) for outcome and covariates. NSS-CES 50 (1994), 55 (2000), and 61 (2005) for MDM coverage data. HAZ height-for-age *z*-score, MDM mid-day meal, SES socioeconomic status. Source data are provided as a Source Data file.

In the birth cohort model, maternal MDM coverage was associated with future child HAZ (Fig. 4a). After adjusting for maternal birth year, wealth, state, and state-specific-birth-year fixed effects, as well as a set of child-specific controls, HAZ in children born to mothers who lived in areas with 100% MDM coverage was 0.40 SD higher than HAZ in children born to mothers living in areas without the MDM (*p* < 0.05). The inclusion of ICDS and PDS access variables did not attenuate this association. The effect of the program varied by SES; children from poor households had the largest effect (0.5 SD, *p* < 0.05) followed by children from middle SES strata (0.33, *p* < 0.05), relative to children from the wealthiest SES strata. In robustness checks, program access coefficients were slightly attenuated but remained significant when adding birth year specific SES fixed effects but were not significant after adding birth year and state-specific SES fixed effects. Further, regressions on subsamples of stunted children showed higher precision but smaller coefficients for the benefits of MDM coverage on HAZ compared to children who were not stunted (Supplementary Fig. 5).

### Controlled interrupted time series analyses

**Fig. 5:**
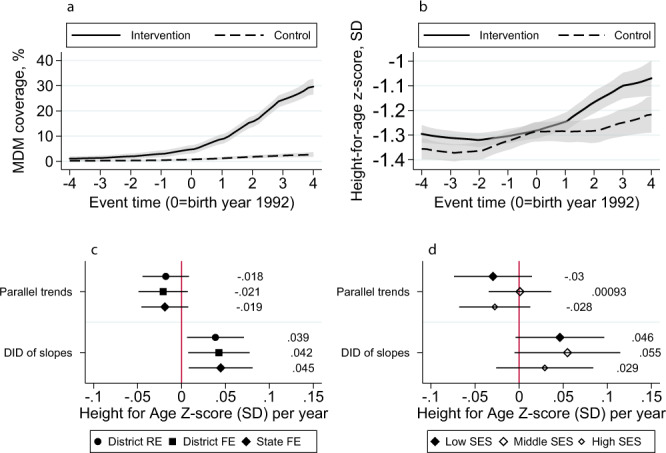
Relationship between MDM and future child HAZ: controlled interrupted time series analyses. All models exclude Kerala and Tamil Nadu. Panel **a** shows MDM coverage by event time across intervention and control states. The program begins between event time 0 and 1. Panel **b** shows the local polynomial of HAZ of children in 2016, born to women belonging to birth cohorts, before and after the start of the program in each state. The shaded gray area indicates the 95% confidence interval. Panel **c** shows the coefficient on γ_6_ (parallel trends) and γ_7_ (DID) from Eq. (3). Coefficients from three models are specified as Eq. (3) plus random effects and fixed effects for district and state. Panel **d**: γ_6_ (parallel trends) and γ_7_ (DID) from Eq. (3) with state fixed effects run on a subset of low (SES 1–3), middle (SES 4–6), and high (SES 7–10) households. The squares/diamonds represent the point estimate and whiskers are 95% confidence intervals. The DID coefficient can be interpreted as the difference in the average rate of change in HAZ, per-year, before versus after MDM started, in the intervention compared to control states All models control for child age, sex, birth order, maternal antenatal care (4+ visits), institutional birth, residence (urban/rural), religion, caste, access to services from the Integrated Child Development Services (dummies for receiving take home rations, child health check-ups, pre-school education, weight measurements, and nutrition counseling) and the Public Distribution System (household has a Below Poverty Line card to obtain subsidized food). All models cluster standard error estimates at the state level. Sources: NFHS 4 (2016) for outcome and covariates. NSS-CES 50 (1994), 55 (2000), and 61 (2005) for MDM coverage data. FE fixed effects, MDM mid-day meal, RE random effects, SES socioeconomic status. Source data are provided as a Source Data file.

The controlled interrupted time series model exploits variation in the timing of the expansion of the MDM program to estimate program benefits relative to a reference period (event time 0 = birth year 1992). MDM expansion between event time 0 and 4 (birth years 1992–1996 capturing the short run impact of the program) differed substantially across intervention and control states (Fig. 5a). Trends in child HAZ were parallel between event time −4 and 0 across intervention and control states (Fig. 5b). After event time 0, intervention states saw a larger change in child HAZ compared to control states. In regression models, the coefficient for parallel trends was not significant, confirming that trends in child HAZ were statistically similar across intervention and control states before the intervention (Fig. 5c). The estimated association was similar across all three specifications, 0.038, 0.041, and 0.044 SD per year (*p* < 0.05). Relative to wealthier households, the effect estimate of the MDM in intervention states was larger among poor and middle-income households at 0.044–0.055 SD per year (*p* < 0.10) (Fig. 5d). In robustness checks, effect coefficients were stable when excluding Gujarat, Odisha, and Chhattisgarh (some districts in these states adopted MDM after Tamil Nadu and Kerala) from treatment states (Supplementary Fig. 2).

### Program pathways

When examining factors that the MDM may work through to influence child HAZ, full MDM coverage during primary school years was a meaningful predictor of all factors examined (Table 1). Full MDM coverage predicted 3.9 years of attained maternal education in years, delaying age in years at first birth by 1.6 years, having a fewer (−0.8) children, a higher probability of having at least four antenatal care visits (22%), and giving birth in a medical facility (28%) (all *p* < 0.001). Full MDM coverage predicted higher adult height among direct beneficiaries (0.51 cm) but the association was not statistically significant.

**Table 1:** Relationship between MDM and direct beneficiary education, height, fertility, and health service use in Indian women born between 1980 and 1998.

	Education, years	Height, cm	Age at first birth, years	Children, number	Antenatal care, binary	Institutional birth, binary
Coefficient	3.95	0.51	1.62	−0.80	0.22	0.28
Standard error	(0.46)	(0.36)	(0.20)	(0.07)	(0.03)	(0.02)
*P* value	<0.000	0.163	<0.000	<0.000	<0.000	<0.000
*R*^2^	0.38	0.12	0.32	0.36	0.24	0.15
*N*	218,810	215,812	218,810	218,810	218,528	218,218

Coefficients are from Eq. (1). Point estimates are interpreted as the difference in the outcome due to 100% exposure to the MDM scheme during primary school years. MDM coverage is the proportion of girls born between 1980 and 1998, within state-specific socioeconomic status deciles, who reported receiving at least 10 meals free of cost at school in the previous month. All models control for residence (urban/rural), religion, and caste. The models include fixed effects for mother’s birth year, state, household wealth, and for state-specific mother’s birth year. All models cluster standard error estimates at the district level. Sources: NFHS 4 (2016) for outcome and covariates. NSS-CES 50 (1994), 55 (2000), and 61 (2005) for MDM coverage data. MDM mid-day meal, SES socioeconomic status.

### Regression decomposition

Our findings can be put into context by considering changes in HAZ among children under 5 years of age reported in the National Family Health Surveys. HAZ improved by 0.4 SDs between 2006 and 2016, on average. Using Eq. (4), with an average MDM coverage of 32% in 2004 at the national level (NSS-CES 61) multiplied by the effect size of 0.166 SD (raw data model) to 0.401 SD (smoothed data model), we estimate the MDM explains 0.053–0.128 SD or 13.3–32.1% of average change in HAZ. Using Eq. (5), with an average of 2.6 years of exposure multiplied by the effect size of 0.044 SD per year, we estimate the MDM can explain 0.114 SD or 28.6% of average change in HAZ. The range estimated contributions are similar in magnitude and relatively substantial, considering that HAZ is dependent on a large set of determinants, of which each can individually only explain a small part of total variation in South Asian countries^11^.

### Migration

A possible concern for our estimates is susceptibility to the effects of migration. Since we measure MDM exposure at the state level in the past and associate it with child nutrition in the future, attribution of the estimated role of MDM exposure would be weakened in the presence of substantial migration across states. A recent study allays this concern by providing estimates on migration in India. Although 30% of India’s population has ever migrated, two-thirds are intra-district migrants, more than half of whom are women migrating for marriage^40^. In 2001, only 4% of India’s population migrated across state borders^40^. Therefore, migration is not a major concern for misclassification of treatment status in our models.

### Discordant SES matching between NSS-CES and NFHS

Overall, we find a 78% concordance between expenditure-based SES deciles measured in 2005 and asset-based SES deciles measured in 2012 at the state level using India’s IHDS (Supplementary Fig. 3). Given this 22% discordance, we cannot rule out that our estimates are somewhat biased due to imperfect classification by SES status. However, the degree of bias is likely to be small because mobility across deciles is limited (the IHDS shows that a household generally only moves up by one or two SES deciles over 7 years, if they move at all) and MDM coverage within states does not fluctuate greatly with small increments of SES classes (in 2005, coverage in the IHDS sample ranged between 53 and 62% in the bottom four SES deciles). Moreover, non-differential misclassification as a form of measurement error generally tends to bias estimates towards the null^41^.

### Matching by caste and religion

To test the sensitivity of our estimates to demographic measures of socioeconomic position that are less likely to change over time, we matched MDM coverage by state of residence, caste, and religion. Similar to SES matching, adjusted full maternal MDM coverage using caste and religion matching was associated with an improvement in HAZ among children aged 0–59 months (Supplementary Fig. 4).

### Selection bias from program placement in government schools

Using 2011 data (IHDS-2) we tested whether girls aged 11–17 years in government schools are shorter than those in private schools. We fit a model with state fixed effects that controls for child age, urban residence, occupation, household size, household expenditure, assets, and parental education. We find that girls in government schools are, on average, 0.89 cm shorter than those in private schools (*p* < 0.001). When we add a dummy variable indicating the receipt of MDM during primary school for these girls (identified in IHDS-1), we find that MDM is associated with a higher height of 1.3 cm on average (*p* < 0.001), while government school attendance is associated with 1 cm lower height (*p* < 0.001). This suggests that selection effects from program placement in government schools are likely to bias our estimates downward and that MDM is the driver of higher height among government school beneficiaries.

### Testing fixed effects models with raw MDM coverage data

The MDM coverage estimate from a regression model using Eq. (1) and the raw coverage data is statistically significant but attenuated to 0.166 SD as expected (Supplementary Table 5, model 1). We also specified a second set of regressions using only the 2004 NSS data, and matched MDM coverage by district and SES. Again, we find an attenuated but significant coefficient of 0.115 SD (Supplementary Table 5, model 2) and, as expected, a larger coefficient of 0.189 SD among poor households (*p* < 0.05) (Supplementary Table 5, model 3). As the district level exposure does not have temporal variation by birth year, this model is not directly comparable with the birth cohort model. However, it does demonstrate that MDM coverage variation by district and SES is strongly correlated with HAZ of children of mothers born between 1993 and 1997.

The MDM coverage estimate from a regression model using Eq. (1) and the log-linear smoothed coverage data are statistically significant but attenuated to 0.261 SD (Supplementary Table 6, model 1). However, attenuation here is smaller in magnitude compared to those using raw data. The model using Eq. (2) shows that children from poor households had the largest effect (0.468 SD, *p* < 0.01) followed by children from middle SES strata (0.296, *p* < 0.05), relative to children from the wealthiest SES strata (Supplementary Table 6, model 2).

Overall, we conclude that both the smoothed and raw data models provide evidence of an effect of maternal MDM coverage on child anthropometry, though the size of the effect depends on the preferred model. We have provided evidence that this effect is robust to varying model specifications, and that the effect of MDM coverage is largest among the poorest households. Moreover, the control interrupted time series models do not use smoothed coverage but provide qualitatively similar estimates.

## Discussion

We have shown that investments made in school meals in previous decades were associated with improvements in future child linear growth. The plausibility of this finding is supported by an association between MDM exposure and underlying determinants of child linear growth: women’s education, fertility, and health service use. As the analysis covers a large nationally representative sample of households, the results reflect a program implemented at scale, with all its flaws, and not a pilot program designed to provide proof of concept. This, of course, comes at a cost; we could not follow a randomized cohort of girls from primary school to childbearing. We put the magnitude of the association into context by using regression decomposition to estimate the share of the actual HAZ improvement explained by the predicted MDM effect on HAZ.

While others have examined the effects of school feeding programs on education and nutrition in beneficiaries themselves, to our knowledge our paper is the first to demonstrate an intergenerational transmission of benefits. This finding provides evidence that, when intergenerational effects are considered, the complete benefit of school feeding programs at scale for linear growth is much larger than previously understood. The result that a school feeding program is related to the nutritional status of children in the next generation also has important implications for other transfer programs. The literature generally focuses on investments in nutrition during the 1000-day period to reduce childhood stunting; our findings suggest that intervening during the primary school years can make important contributions to reducing future child stunting, particularly given the cumulative exposure that is possible through school feeding programs.

School meal programs are often motivated by their potential to increase schooling, particularly that of girls. While enrolment parity is within reach in primary schooling –between 2000 and 2015, the number of primary school-age children not in school declined globally from 100 million to 61 million^42^—there is a larger goal of primary and post primary school completion. Very little in the literature on school meal programs can quantify program contribution to total years of schooling completed. Moreover, evidence that the scale-up of school meals is associated with increased heights of women—in a population in which stunting has been historically linked with maternal undernutrition—provides a new perspective on the contribution of such programs. This reinforces an increased attention to seeking opportunities to improve nutrition in the “next 7000 days”^8^, that is, to find means of addressing undernutrition should efforts in the high priority period prior to a child’s second birthday not be fully successful. The results here show that school meals may contribute to education, nutrition (height), later fertility decisions, and access to health care; by doing so, school meals may reduce the risk of undernutrition in the next generation. In its current form, India’s MDM scheme has the potential to address multiple underlying determinants of undernutrition. Improving the quality of meals provided and extending the program beyond primary school might further enhance its benefits^8^, though we could not empirically test these hypotheses given the available data.

The MDM is mandated by the Supreme Court of India as a social protection program addressing food insecurity. The social protection role of addressing hunger and food insecurity may be a justification by itself for school-based transfers in many settings^43^. However, evidence such as presented here depict these programs as contributing to both food security and to improved outcomes in the next generation, thus contribute to the policy framework for school-based interventions.

## Methods

### Data sources

This paper relies on evidence from seven rounds of three publicly available nationally representative surveys (Supplementary Table 2). The primary analysis in this paper uses data on whether children born between 1980 and 1998 received free meals at school from the National Sample Survey of Consumer Expenditure (NSS-CES) (1993, 1999, and 2004 rounds)^44–47^. These data are combined with data on child height-for-age *z*-scores in 2016 from wave four of India’s Demographic Health Survey, the National Family Health Survey (NFHS)^48^. Both are large nationally representative surveys, which make it possible to match exposure to MDM by cohorts of girls born between 1980 and 1998 at the district level with data on mean child height in the same locations in 2016. The 2016 NFHS4 sample included 217,940 women with 196,310 children under 5 years of age. NSS-CES data from 2011 were also used for generating maps for coverage but not for the primary analyses. Our interest was examining next generation benefits on child stunting and our hypothesis was that intergenerational effects work through first generation improvements in education, height, fertility, and access to health services^14,43,49–52^. We expected larger influence of maternal coverage compared to paternal coverage given previous evidence showing larger program impacts on girls than on boys^18^. We support our main findings by using the 2004 and 2011 rounds of Indian Human Development Surveys for descriptive analyses and robustness checks^53,54^. IHDS provides a wide array of variables that are not available in the NSS or the NFHS and offers supportive evidence on the main estimates and model assumptions. Our study was a secondary analysis of existing public survey data; hence, no ethical approval was required for our study. All surveys complied with ethical norms with appropriate approvals and consent taken at the time of survey. Summary statistics for the primary and secondary outcomes examined in this paper are shown in Supplementary Table 3. Summary statistics for the covariates from NFHS are shown in Supplementary Table 4.

### Identification strategy

In an ideal experiment, children would be randomly assigned access to free lunches from the MDM program in primary school and we would compare the average HAZ outcomes for the children of the MDM beneficiaries and of the MDM non-beneficiaries when the original children in the experiment reached adulthood. In the absence of randomized treatment allotment, we chose to use panel data techniques from repeated cross-sections^55^ to exploit the strengths of the available data for identification—the fact that the data cover birth cohorts over a long period and that MDM coverage varies by state of residence and SES. SES was calculated using a principal component analysis of household assets, including cooking fuel, floor and wall materials, land and house ownership; and the possession of assets, including a mattress, pressure cooker, chair, bed, table, fan, TV, sewing machine, phone, computer, fridge, watch, bicycle, motorbike, car; and the possession of animals, including cows, goats, and chickens.

### Birth cohort fixed effects analyses

Year of birth, SES decile, and state of residence were used to determine an individual’s exposure to the program. In India, children are expected to attend primary school between the ages of 6 and 10 years. The NSS-CES provide data on the age of all household members and whether they received free meals at school in the past 30 days. Of all the girls aged 6–10 years in the 2005 NSS sample who reported receiving any free meals at school (*N* = 8873), 95.6% reported receiving at least 10 meals in the previous month. We used a minimum of 10 meals per month to ensure that our coverage estimates were for children who received the program with fidelity. Models were run separately using any MDM access (at least 1 meal) and comparable results were obtained. We use this information to calculate the percentage of all girls aged 6–10 years covered by the program for cohorts born between 1980 and 1998. This period gives us an approximately equal number of birth cohorts who were born before and after the introduction of the MDM scheme. Since the MDM scheme was introduced in 1995, those born after 1989 would be able to receive free meals in primary school. In addition, the NSS-CES provide measures of SES and state of residence, which allowed us to calculate coverage rates for all girls aged 6–10 years, specific to each SES strata in all Indian states.

For any cohort, MDM exposure is a function of the number of years an average child spends in primary school and when the program started in the school they attended. In an ideal data setting, to obtain an accurate coverage estimate for a birth cohort, we would have data from five cross-sections surveyed consecutively. For example, to obtain an estimate of MDM coverage for the 1994 cohort, we ideally would have coverage data on 6-year-old children measured in 2000, 7-year-olds in 2001, 8-year-olds in 2002, 9-year-olds in 2003, and 10-year-olds in 2004. We would then average these five coverage estimates into a single estimate, representing average MDM exposure assuming a typical five-year period in primary school for the 1994 cohort. The averaging is necessary because any single year does not accurately reflect exposure for all 5 years in primary school.

Each NSS-CES repeated cross-section, conducted within 5 year intervals, provides MDM coverage by child age as measured in the survey year. We used linear interpolation to estimate a smoothed continuous exposure indicator that varies by maternal birth year, state, and SES. For example, using coverage estimates for 6 year olds in 1999 NSS-CES (birth year 1993) and 6 year olds in the 2004 NSS-CES (birth year 1998), we first used linear interpolation to estimate the average rate of increase in MDM coverage for 6 year olds for the years (2000, 2001, 2002, and 2003) with no NSS-CES data (these correspond with birth years 1994, 1995, 1996, and 1997). Next, we performed similar interpolation for 7-, 8-, 9-, and 10-year-old children. This provided smoothed coverage estimates for children born in 1993 for the survey years 1999, 2000, 2001, 2002, and 2003—the years the 1993 cohort would have aged from 6 to 10 years. We take the average coverage for these 5 years as the final estimate of coverage experience of a specific birth year (Supplementary Fig. 1). This process of smoothing (i) estimates the relationship between maternal school meals exposure and annual child HAZ outcomes under an assumption of a linear trend in exposure and (ii) reduces probable bias due to measurement error present in the raw data by moving extreme values closer to the center of the distribution.

Throughout the paper, we use the term MDM coverage, which refers to an estimate of state-by-year average program exposure during primary school for the birth cohorts in the sample, under the assumption that coverage increases in a linear fashion within age groups of children in primary school surveyed in the years 1993, 1999, 2004, and 2011. It is almost certain that exposure in the interval between 2 years lies between the values in the end points; the assumption that the expansion is linear is a plausible pattern of program roll out. We assume that within 5-year intervals, the duration of primary school, age-specific trends in coverage would have increased gradually. Gradual rollout is typical of at-scale programs in developing countries with numerous implementation, financing, and bureaucratic challenges^56^. However, in sensitivity analyses, we subject the assumption of a linear scale-up to an additional robustness check where we smooth MDM coverage using a log-linear process.

Next, using birth year, SES deciles and state of residence, we match NFHS data with NSS-CES data for the percentage of girls covered by the MDM for cohorts born between 1980 and 1998. NFHS data provide anthropometric measurements for the last three births for each mother. We use data for all available children with valid anthropometric measurements. We calculate HAZ using the “zscore06” STATA routine which automatically excludes outlier measurements.

We specify the following model:

1 $${{{Y}}}_{{{iwst}}}={\gamma }_{0}+{\gamma }_{1}{{\rm{MDM}}}_{{{wst}}}+{\gamma }_{2}{{\bf{T}}}_{{{t}}}+{\gamma }_{3}{{\bf{S}}}_{{{s}}}+{\gamma }_{4}{{\bf{S}}}_{{{s}}} * {{\bf{T}}}_{{{t}}}+{\gamma }_{5}{{\bf{W}}}_{{{w}}}+{\gamma }_{6}{{\bf{C}}}_{{{iwst}}}+{\gamma }_{7}{{\bf{P}}}_{{{iwst}}}+{{\rm{\varepsilon }}}_{{{iwst}}}$$

where *Y*_*iwst*_ is the height-for-age *z*-score for child *i* belonging to SES strata *w* in state *s* in mother’s birth year *t*. MDM_*wst*_ is a continuous indicator coded as the proportion of mothers covered by the MDM as children and ranges between 0 and 1. **T**_*t*_ represents birth-year fixed effects which forces identification of within birth-year effects and controls for time-varying national level economic changes, programs, and policies. Examples of these are national programs such as the National Health Mission introduced in 2005 (ref. ^57^) and changes in national GDP, which has shown robust growth^58^.

We estimated Eq. (1) using MDM coverage at the state level disaggregated by wealth strata. $\,{{\bf{W}}}_{w}$ represents the wealth-decile fixed effects and provides controls for all unobserved time-invariant factors associated with household wealth and MDM coverage. **S**_***s***_ is the state fixed effects which controls for all for time-invariant differences across states with high and low MDM exposure. **S**_*s*_ *  **T**_*t*_ or state-birth-year fixed effects controls for unobserved state-specific time-varying factors that could be correlated with the outcome such as the state’s political climate, varying degrees of implementation of welfare programs, agricultural policies, and educational subsidies. A concern for a model estimated without this parameter is that states that introduced free meals in primary school at different times and rates of coverage expansion could be systematically different. For example, states with residents who had lower education or poorer nutritional status on average may have been more likely to introduce the MDM. Similarly, states with better governance may have been better equipped to implement the MDM program at scale. In either case, the correlation between outcomes and MDM implementation could be confounded with unobserved state-specific time-varying factors.

**C**_*iwst*_ represents a vector of individual, household and survey-specific controls, including child age, sex, birth order, mothers antenatal care status during pregnancy, birth in a medical facility, and household characteristics at the time the outcome was measured. The vector includes SES, caste, religion, and residence (urban or rural). **P**_*iwst*_ represents a vector of individual and household-specific programmatic controls, including access to services from the Integrated Child Development Services (dummies for receiving take home rations, child health check-ups, pre-school education, weight measurements, and nutrition counseling) and the Public Distribution System (household has a Below Poverty Line card to obtain subsidized food)^59,60^. Controlling for these variables reduces possible confounding from government interventions that could benefit current child nutritional status. All standard error estimates were clustered at the district level. Clustering adjusts standard error estimates after accounting for intra-district correlations and assumes that residuals are independent across districts^61^.

The coefficients estimated by Eq. (1) are intent-to-treat (ITT) estimates because the MDM coverage variable measures “potential exposure” to the program on entire birth cohorts. Our ITT estimates are a policy-relevant parameter for an ex-post analysis of the effects of a large program on the entire population (birth cohorts)^21,22^. Our models, based on population representative MDM coverage, estimate the magnitude of improvement in child undernutrition that can be expected if a cohort is potentially treated.

### Testing for differential benefits for the poor

IHDS data show that 80% of all MDM beneficiaries in 2004 attended government schools and that two-thirds of children attending government schools were from low-income households (bottom six SES deciles), suggesting that MDM was primarily implemented in government schools rather than in private schools as an incentive for children from poor households to attend primary school (and to improve nutrition); therefore, the estimates in Eq. (1) are likely to mask heterogeneity of response to the program. Masking is anticipated because outcome data from children sampled from non-poor households, who would be more likely to opt out of the government school system in favor of private schools, would influence average effect sizes^62^. We expect that mothers who were enrolled in government schools during their childhood would have worse nutritional outcomes and this might place a downward bias on our estimates. To investigate the existence of such heterogeneity, we compared associations across SES groups. We created SES deciles and grouped women in the bottom three (poor), middle three, and top four (non-poor) deciles to create two wealth strata. We estimated models for differential associations for poor, middle versus non-poor households by modifying Eq. (1) as follows:

2 $${{{Y}}}_{{{iwst}}} ={\gamma }_{0}+{\gamma }_{1}{{\rm{MDM}}}_{{{wst}}} * {{\rm{Poor}}}_{{{wst}}}+{\gamma }_{2}{{\rm{MDM}}}_{{{wst}}} * {{\rm{Middle}}}_{{{wst}}}+{\gamma }_{3}{{\rm{MDM}}}_{{{wst}}}\\ \quad +\,{\gamma }_{4}{{\bf{T}}}_{{{t}}}+{\gamma }_{5}{{\bf{S}}}_{{{s}}}+{\gamma }_{6}{{\bf{S}}}_{{{s}}} * {{\bf{T}}}_{{{t}}}+{\gamma }_{7}{{\bf{W}}}_{{{w}}}+{\gamma }_{8}{{\bf{C}}}_{{{iwst}}}+{\gamma }_{9}{{\bf{P}}}_{{{iwst}}}+{{\rm{\varepsilon }}}_{{{iwst}}}$$

where ${{\rm{Poor}}}_{{{wst}}}$ and Middle_*wst*_ dummy variables for bottom three and middle (4–6) SES deciles, respectively, with the top four SES deciles serving as the reference non-poor group. ${\gamma }_{1}$ and ${\gamma }_{2}$ measure if poor and middle SES households benefitted more from MDM coverage compared to non-poor households. We expect ${\gamma }_{1}$ to be larger than ${\gamma }_{2}$, and if these coefficients are statistically significant and of a large order, then we have evidence that MDM program benefits differ by SES. Note that SES here is current, and mother’s SES may have differed in childhood. To this end, we offer evidence in our sensitivity analyses that SES mobility is likely modest.

#### Controlled interrupted time series models using state rollout timing

The birth cohort model exploits variation in treatment measured as the proportion of children covered by the program within a birth year, state, and across SES strata. It allows us to express the relationship between MDM and HAZ as a function of coverage. However, it comes at the cost of potential for endogeneity because MDM coverage could potentially be associated with changes in living conditions that vary within cohorts defined by state, birth year, and SES strata. An alternate model exploits the differential timing of MDM rollout across Indian states as a robustness check on the birth cohort model. This alternative can reveal insights for the short-term cumulative benefits of the program^31^.

States implemented the program at different times; de-facto, the program was rolled out in the three phases (Supplementary Table 1). According to the NSS data, MDM coverage patterns by state and birth year show that Tamil Nadu and Kerala, i.e. “phase 1” states, had average coverage greater than 20% for maternal birth year 1988). These states initiated school feeding programs well before the central government funded MDM. Following the central government order, in phase 2, other states—Odisha, Himachal Pradesh, Uttaranchal, Haryana, Rajasthan, Sikkim, Tripura, West Bengal, Chhattisgarh, Madhya Pradesh, Gujarat, Maharashtra, Andhra Pradesh, and Karnataka—implemented the program at scale with coverage increasing by more than 10% between maternal birth years 1992–1996. In the remaining states (phase 3), MDM coverage was below 5% and increased by less than 10% between maternal birth years 1992–1996.

These roll-out patterns lend themselves to analysis using a controlled interrupted time series design (CITS)^63–65^. Conceptually, the CITS is a combination of the difference-in-differences and interrupted time series models. It includes a within group before–after comparison, and a between-group comparison, strengthening the control for potential confounders. The first difference is the change in the outcome trend within each group, comparing the period before MDM to the period after (slope change). The second difference is the difference in slope changes in the control group compared to the intervention group (difference-in-differences of slopes). The CITS reduces bias due to other interventions or events occurring around the same time as the MDM intervention and allows comparison groups to start at different levels of the outcome. Moreover, the CITS controls for the improvement in HAZ that would be expected without the MDM and tests for parallel trends within the model.

We exclude Tamil Nadu and Kerala from CITS analysis as they were early MDM implementers and both states have better nutrition outcomes compared to other states in India. We focus on maternal birth years 1988 to 1996, when we have a pre intervention period with no MDM across all states, and a post intervention period when some states introduced the program while others did not. Phase 2 states form the intervention group and phase 3 states serve as the control group. We parameterize the CITS model using Eq. (3).

3 $${{{Y}}}_{{{ist}}} ={\gamma }_{0}+{\gamma }_{1}{{\rm{Int}}}_{{{s}}}+{\gamma }_{2}{{\rm{T}}}_{{{t}}}+{\gamma }_{3}{{\rm{Post}}}_{{{t}}}+{\gamma }_{4}{{\rm{Post}}}_{{{t}}} * {{\rm{T}}}_{{{t}}}+{\gamma }_{5}{{\rm{Post}}}_{{{t}}} * {{\rm{Int}}}_{{{t}}} \\ \quad +\,{\gamma }_{6}{{\rm{T}}}_{{{t}}} * {{\rm{Int}}}_{{{s}}}+{\gamma }_{7}{{\rm{T}}}_{{{t}}} * {{\rm{Int}}}_{{{s}}} * {{\rm{Post}}}_{{{t}}}+{\gamma }_{8}{{\bf{C}}}_{{{ist}}}+{\gamma }_{9}{{\bf{P}}}_{{{ist}}}+{{\rm{\varepsilon }}}_{{{ist}}}$$

In state *s* in mother’s birth year *t*, ${{\rm{Int}}}_{{{s}}}$ is a dummy for the intervention states, ${{\rm{T}}}_{{{t}}}$ is the event time, a discrete variable (for maternal birth years 1988–1996) that is centered at 1992 and ranges between −4 and 4. ${{\rm{Post}}}_{{{t}}}$ is a dummy for maternal birth years 1993–1996. In Eq. (3), ${\gamma }_{6}$ tests the null hypothesis of parallel pre-intervention trends; if not significant, we reject this hypothesis and conclude that pre-interventions differed between intervention and control groups. ${\gamma }_{7}$ is the coefficient of interest, and represents a “difference-in-difference of slopes” between the intervention and control states. If ${\gamma }_{7}$ is statistically significant, the change in HAZ slope for intervention states differs from the change in HAZ slope for control states. In other words, it tests for faster gains in child linear growth for states with MDM. To account for spatial heterogeneity, we run three specifications of Eq. (3) by adding district random effects, district fixed effects, and state fixed effects.

To explore heterogeneity, we investigate differential associations by household SES by running models within subsamples of poor (SES deciles 1–3), middle (SES deciles 4–6), and non-poor (SES deciles 7–10) households. For robustness, we check sensitivity of coefficients to exclusion of Gujarat, Odisha, and Chhattisgarh from the intervention group. These states had greater than 10% coverage at event time 0 and thus could arguably be placed in the phase 1 category.

### Regression decomposition

To test the plausibility of our results we performed regression-based decomposition with our estimates from Eqs. (1) and (3) (ref. ^66^). From Eq. (1), we estimated the population level effect of the program between 2006 and 2016 with Eq. (4).

4 $$\frac{\prod {\gamma }_{1}{{\mathrm{{MDM2004}}}}\,}{\Delta {\mathrm{{HAZ}}}}$$

where ${\gamma }_{1}$ is the coefficient of MDM from Eq. (1), MDM_2004_ is the MDM coverage in 2004 and ΔHAZ change in HAZ between 2006 and 2016.

From the controlled interrupted time series model, we estimated the effect of exposure to the program using Eq. (5).

5 $$\frac{\prod {\gamma }_{7}{{\mathrm{{PostEventTime}}}}\,}{\Delta {\mathrm{{HAZ}}}}$$

where ${\gamma }_{7}$ is the coefficient from Eq. (3), ${{\mathrm{{PostEventTime}}}}$ is the average event time 5 years before and after the start of the program, and ΔHAZ is the change in HAZ between 2006 and 2016. Estimates from Eqs. (4) and (5) are proportions and are expected to be less than 1 because the predicted difference in HAZ explained by MDM must be less than the total change in HAZ observed between 2004 and 2016.

### Program pathways

We next investigated plausible pathways that might support intergenerational links between the MDM program and child nutrition. We used Eq. (1) to investigate the association of the MDM with six factors that may be related to the MDM program and which, in turn, correlate with child HAZ: mother’s education and height, mother’s age at first birth, total number of children per mother, number of antenatal care visits attended by the mother during pregnancy and if the child was born in a medical facility. We recognize that this is a plausibility analysis and cannot isolate causality.

### Discordant SES matching between NSS-CES and NFHS

We matched MDM coverage by state of residence and SES decile between the NSS-CES and NFHS. This assumes that (1) mobility across SES strata over time is minimal and (2) SES deciles in NFHS correspond well with those in the NSS. We therefore use panel data from the IHDS to assess the concordance of expenditure-based SES deciles measured in 2005 and asset-based SES deciles measured in 2012. Since IHDS follows the same individuals over 7 years, we can track their mobility across SES strata over time and then compare their status on both SES measurements.

### Matching by caste or religious group

To test the sensitivity of our estimates to demographic measures of socioeconomic position, we matched maternal MDM coverage by birth year, state of residence and households’ caste/religious groups in the NSS-CES and NFHS. The social groups used to match households were scheduled caste (Hindu), scheduled tribe (Hindu), Muslim, Christian, and others. Similar to the SES model, this model works by assigning a probability of exposure to MDM for maternal birth cohorts that varies by state, religion, and caste. While social groups do not follow strict income hierarchies across states, they have the advantage of being largely time invariant and thus do not introduce biases that result from income mobility.

### Testing fixed effects models with raw MDM coverage data

To test the sensitivity of our estimates using smoothed coverage, we offer an additional alternative using raw coverage data from NSS. These coverage estimates are from cross-sections at specific points in time and are not smoothed using the age profiles of children in the NSS rounds. We first created a scatter plot of smoothed coverage estimates against the raw coverage data to gauge the degree and direction of the smoothing process (Supplementary Fig. 6).

The maps in Fig. 1 show a discrete jump in coverage from 6% in 1999 to 32% in 2004. The smoothed data attempt to fill in data gaps on coverage for the years 2000 to 2003. The scatterplot of the smoothed coverage data against the raw data shows that the smoothed data are less extreme than the raw data, which has many 0 and 100% coverage estimates. These extreme values present in the raw data likely reflect measurement error for cohort-specific coverage because they do not capture the transition of increasing coverage for the initial years of program implementation, so that coverage for any observation reflects only a single year during a time of program expansion despite the fact that a student will have spent more than a single year in school. To test our hypothesis that measurement error in the raw coverage data would attenuate results compared to those from the models using smoothed data in keeping with standard expectation with random errors in variables, we ran our primary birth cohort model with raw coverage matched by state, SES, and birth years. We also ran a second test of sensitivity with models that use only the 2004 NSS raw coverage data, matched by district and SES. These models specify the same level of coverage to SES groups within districts for birth cohorts 1993 to 1997.

### Testing fixed effects models with MDM coverage smoothed using log-linear process

To test the sensitivity of our estimates using linearly smoothed coverage, we use an alternative log-linear smoothing process. This process assumes an exponential growth in MDM coverage within 5-year intervals. We then fit models with Eqs. (1) and (2).

### Reporting summary

Further information on research design is available in the Nature Research Reporting Summary linked to this article.

## Supplementary information

Supplementary Information

Peer Review File

Reporting Summary

## Data Availability

The conclusions of this article are based on publicly available datasets^44–48,53,54^. Source data are provided with this paper. The cleaned and merged dataset is available on the Harvard Dataverse at [10.7910/DVN/JTN87W]^67^. Source data are provided with this paper.

## Source data

Source Data

## Code availability

The analysis code that reproduces all the tables and figures in the manuscript is available on the Harvard Dataverse at [10.7910/DVN/JTN87W]^67^.
