# Supplementary material for: Intergenerational nutrition benefits of India’s national school feeding program
Source: Nat Commun. 2021 Jul 12;12:4248. doi: 10.1038/s41467-021-24433-w (PMC8275765; doi:10.1038/s41467-021-24433-w)
Supplement: Supplementary file 1 — Supplementary Information [file 41467_2021_24433_MOESM1_ESM.pdf]

SUPPLEMENTARY INFORMATION

Table of Contents

Supplementary Table 1. Mid-day meal program rollout: percent of girls age 6-10 years receiving the program, by state and maternal birth year ... 2

Supplementary Table 2. Summary of datasets used to investigate the intergenerational effect of India's mid-day meal program on child stunting ... 3

Supplementary Table 3. Summary statistics for outcome variables in Indian children under five years of age in 2016, and in women born between 1980 and 1998. ... 4

Supplementary Table 4. Summary statistics of the National Family Health Survey sample 2016 ... 5

Supplementary Table 5. Regression models using raw data on MDM coverage ... 6

Supplementary Table 6. Regression models using MDM coverage data smoothed with log-linear interpolation ... 7

Supplementary Figure 1: Coverage of Mid-Day Meal program by state and maternal birth year ... 8

Supplementary Figure 2: Sensitivity analyses for controlled interrupted time series model ... 9

Supplementary Figure 3: State level correlation between deciles of monthly per-capita expenditure measured in 2005 and asset-based deciles in 2012. ... 10

Supplementary Figure 4. Matching exposure to MDM by caste and religious groups instead of SES ... 11

Supplementary Figure 5. Heterogeneity in program benefits among children who differ by height ... 12

Supplementary Figure 6. Correspondence between raw MDM coverage data and interpolated smoothed coverage data ... 13

Supplementary Table 1. Mid-day meal program rollout: percent of girls age 6-10 years receiving the program, by state and maternal birth year

	Maternal birth year		
	**1988**	**1992**	**1996**
	**Phase 1**		
TAMIL NADU	39	48	57
KERALA	22	39	51
	**Phase 2**		
GUJARAT	9	14	36
ORISSA	3	20	30
CHATTISGARH	2	16	45
TRIPURA	0	1	50
HIMACHAL PRADESH	0	4	45
SIKKIM	0	1	42
UTTARANCHAL	0	1	37
KARNATAKA	0	1	37
MAHARASTRA	0	1	34
ANDHRA PRADESH	0	2	30
MADHYA PRADESH	0	3	26
WEST BENGAL	0	2	23
HARYANA	0	1	19
RAJASTHAN	0	1	14
	**Phase 3**		
ARUNACHAL PRADESH	1	4	11
JHARKHAND	0	1	9
DELHI	0	1	9
UTTAR PRADESH	0	1	8
BIHAR	0	0	4
ASSAM	0	0	3
PUNJAB	0	0	1
NAGALAND	0	0	1
MANIPUR	0	0	0
MIZORAM	1	0	0
MEGHALAYA	0	0	0
GOA	0	0	0

Reported numbers are percent of girls age 6-10 years covered by MDM in a state.
Source: NSS-CES 50 (1993-94), 55 (1999-2000), 61 (2004-2005) and 68 (2011-2012) for MDM coverage data.

Supplementary Table 2. Summary of datasets used to investigate the intergenerational effect of India's mid-day meal program on child stunting

Data source	Survey rounds used	Survey Type	Variables used
**Primary analysis**			
National Sample Surveys of Consumer Expenditure (NSS-CES)	1993-1994 (NSS-CES50) 1999-2000 (NSS-CES55) 2004-2005 (NSS-CES61) 2011-2012 (NSS-CES68)	Repeated cross-section at individual level, representative at state and country level. Two-stage sample design selected with probability proportional to size followed by random sampling, separately for urban and rural areas.	Mid-day meal program participation among girls and boys by birth year
National Family Health Survey (NFHS)	2015-2016 (NFHS4)	Cross-section at individual level, representative at state level and district level. Two-stage sample design selected with probability proportional to size followed by random sampling, separately for urban and rural areas.	Height-for-age z-score, child age, birth order and sex, mother's age, height, education, and socio-economic status[1].
**Secondary analysis**			
Indian Human Development Survey (IHDS)	2004-2005 (IHDS1) 2011-2012 (IHDS2)	Panel at individual level, representative at country level. Revisited sample of 1994 Human Development Profile of India (HDPI) Survey. A three-stage sample was randomly drawn in each of new states or territories not covered in HDPI. Attrition between 2004-05 and 2011-12 rounds replaced by refresher sample randomly drawn with probability proportional to population size.	Mid-day meal program participation among girls aged 6-10 years, and socioeconomic variables[2]

[1]Index of socio-economic status (SES) was constructed by conducting factor analysis using: household access to improved drinking water, improved latrine, clean cooking fuel, electricity and possession of durable household assets including a mattress, pressure cooker, chair, bed, table, fan, tv, sewing machine, phone; and housing materials for floor, roof and wall, if the household owned land or owned a house
[2]government school, monthly consumption expenditure per-capita (INR), household assets (number) and state of residence .

Supplementary Table 3. Summary statistics for outcome variables in Indian children under five years of age in 2016, and in women born between 1980 and 1998.

	N	Mean/proportion	95% CI
Primary outcome			
Height-for-age z-score, SD	197,088	-1.38	-1.39, -1.36
Secondary outcomes			
Women's education, years	218,810	6.62	6.59,6.65
Women's height, cm	215,812	151.65	151.61,151.68
Age at first birth, years	218,810	20.77	20.75,20.80
# of children per woman	218,810	2.23	2.22,2.23
4+ Antenatal care visits, binary	218,528	0.50	0.50,0.50
Institutional birth, binary	218,218	0.80	0.80,0.81

Sources: National Family Health Survey 4 (2016). Estimates are produced using household sampling weights to render the means and proportions representative at the national level.

Supplementary Table 4. Summary statistics of the National Family Health Survey sample 2016

	Mean / proportion	*95% CI*
Mothers		
N	218,810	
Mother's age, yr	26.17	[26.14,26.19]
Children		
N	207,345	
Male child, binary	0.53	[0.53,0.53]
Child age, months	29.10	[28.99,29.21]
Child birth order		
First born	0.39	[0.38,0.39]
Second born	0.34	[0.34,0.34]
Third born	0.16	[0.16,0.16]
Households		
N	209,765	
Wealth score		
Quintile 1 (Poorest)	0.25	[0.25,0.25]
Quintile 2	0.21	[0.21,0.22]
Quintile 3	0.20	[0.20,0.20]
Quintile 4	0.20	[0.20,0.20]
Quintile 5 (Non-poor)	0.13	[0.13,0.14]
Household characteristics		
Improved drinking water, binary	0.90	[0.89,0.90]
No toilet facility, binary	0.45	[0.45,0.46]
Clean fuel for cooking, binary	0.36	[0.35,0.36]
Owns house, binary	0.80	[0.80,0.80]
Kachha house, binary	0.07	[0.07,0.07]
Semi-pucca house, binary	0.42	[0.42,0.42]
Pucca, binary	0.51	[0.51,0.51]
Floor		
Mud/clay/earth/sand/dung/planks/bamboo	0.42	[0.42,0.42]
Brick/stone/strips	0.06	[0.06,0.06]
Polished wood/tiles/cement/carpet/granite	0.52	[0.52,0.53]
Roof		
None/leaf/mud/grass/mat/bamboo/unburnt Bricks/timber/loosely packed stone	0.16	[0.16,0.16]
Polythene/metal/wood/asbestos sheets/roofing shingles	0.24	[0.24,0.24]
Concrete/tiles/slate/burnt brick	0.60	[0.60,0.61]
Wall		
None/palm/mud/grass/bamboo/stone/ plywood/cardboard/unburrnt brick/raw wood	0.26	[0.25,0.26]
Shingles/asbestos sheets	0.74	[0.74,0.75]
Social group		
Scheduled castes, binary	0.22	[0.22,0.22]
Scheduled tribe, binary	0.10	[0.10,0.10]

Sources: National Family Health Survey 4 (2016). Estimates are produced using household sampling weights to render the means and proportions representative at the national level.

Supplementary Table 5. Regression models using raw data on MDM coverage

	(1) State, Birth-year & SES matched			(2) District & SES matched			(3) District & SES matched		
	**Beta**	**SE**	**p**	**Beta**	**SE**	**p**	**Beta**	**SE**	**p**
MDM coverage	0.166	(0.072)	0.021	0.115	(0.051)	0.027	0.005	(0.081)	0.951
MDM x SES 1-3							0.189	(0.092)	0.041
MDM x SES 4-6							0.093	(0.088)	0.291
Maternal birth years	1984-1997			1993-1997			1993-1997		
Birth year fixed effects	Yes			Yes			Yes		
SES fixed effects	Yes			Yes			Yes		
State fixed effects	Yes			Yes			Yes		
State x Birth year fixed effects	Yes			No			No		
District random effects	No			Yes			Yes		
NSS CES rounds used	1993,1999,2004			2004			2004		
N	168,367			30,576			30,576		

Standard errors estimates clustered by district in parentheses. P values are calculated from two tailed regression based t tests. Corrections for multiple comparisons were not made in these tests because only one outcome is being studied in all regressions. All models control for child age, sex, birth order, maternal antenatal care (4+ visits), institutional birth, residence (urban/rural), religion, caste, access to services from the Integrated Child Development Services (dummies for receiving take home rations, child health check-ups, pre-school education, weight measurements, and nutrition counseling) and the Public Distribution System (household has a Below Poverty Line card to obtain subsidized food). MDM=Mid-day meal; SES=Socio-economic status, NSS CES=national sample survey of consumer expenditure

Supplementary Table 6. Regression models using MDM coverage data smoothed with log-linear interpolation

	Model 1			Model 2		
	**Beta**	**SE**	**p**	**Beta**	**SE**	**p**
MDM coverage	0.261	(0.093)	0.005	-0.257	(0.132)	0.053
MDM x SES 1-3				0.468	(0.106)	0.000
MDM x SES 4-6				0.296	(0.120)	0.014
Birth year fixed effects	Yes			Yes		
SES fixed effects	Yes			Yes		
State fixed effects	Yes			Yes		
State x Birth year fixed effects	Yes			Yes		
NSS CES rounds used	1993,1999,2004			1993,1999,2004		
N	196,386			196,386		

Standard errors estimates clustered by district in parentheses. P values are calculated from two tailed regression based t tests. Corrections for multiple comparisons were not made in these tests because only one outcome is being studied in all regressions. All models control for child age, sex, birth order, maternal antenatal care (4+ visits), institutional birth, residence (urban/rural), religion, caste, access to services from the Integrated Child Development Services (dummies for receiving take home rations, child health check-ups, pre-school education, weight measurements, and nutrition counseling) and the Public Distribution System (household has a Below Poverty Line card to obtain subsidized food). MDM=Mid-day meal; SES=Socio-economic status, NSS CES=national sample survey of consumer expenditure

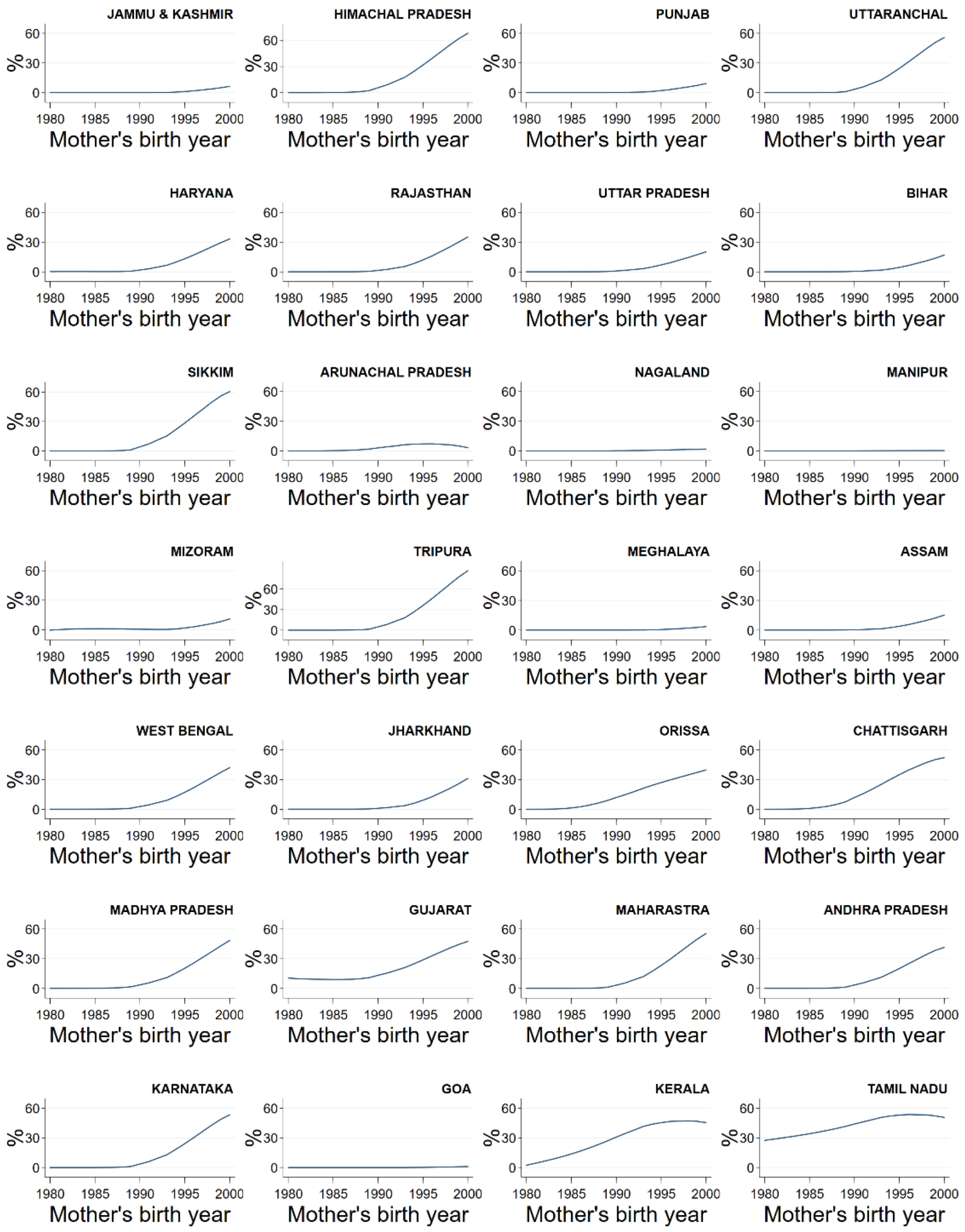

Supplementary Figure 1: Coverage of Mid-Day Meal program by state and maternal birth year

Reported numbers are percent of girls covered by MDM in a state.
Source: NSS-CES 50 (1993-94), 55 (1999-2000), 61 (2004-2005) and 68 (2011-2012) for MDM coverage data. Source data are provided as a Source Data file.

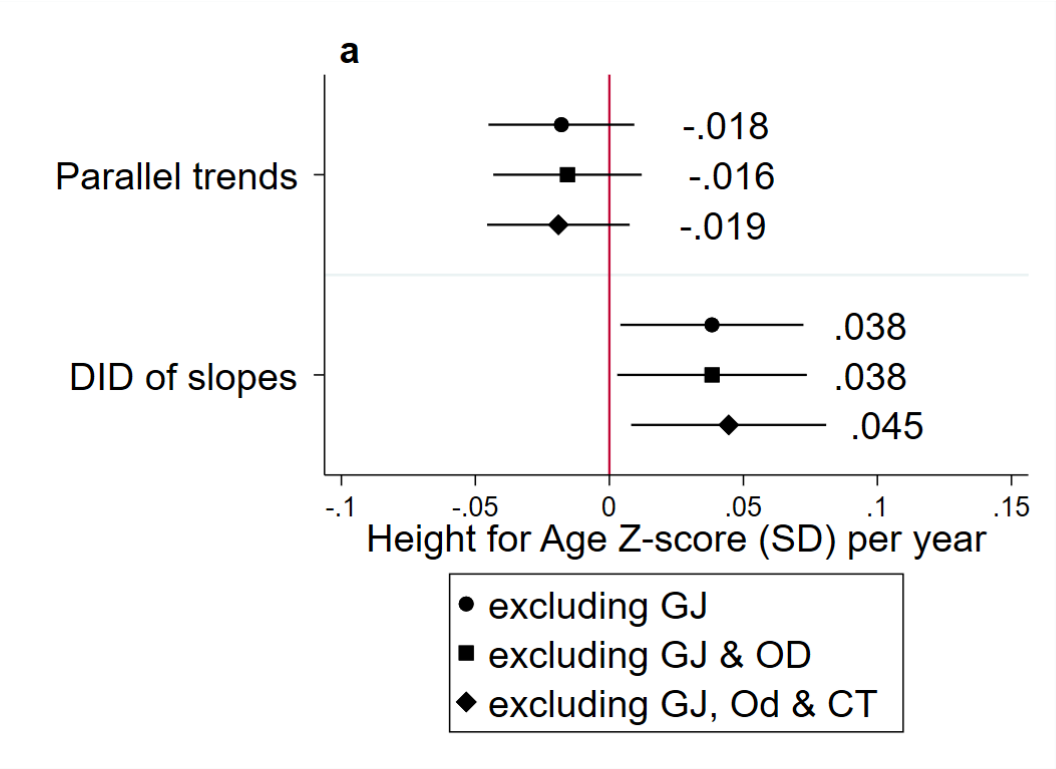

Supplementary Figure 2: Sensitivity analyses for controlled interrupted time series model

Panel A shows the coefficient on γ_6 (parallel trends) and γ_7 (DID) from equation 3. The shapes represent the point estimate and whiskers are 95% confidence intervals. The DID coefficient can be interpreted as the difference in the average rate of change in HAZ, per-year, before versus after MDM started, in the intervention compared to control states All models control for child age, sex, birth order, maternal antenatal care (4+ visits), institutional birth, residence (urban/rural), religion, caste, access to services from the Integrated Child Development Services (dummies for receiving take home rations, child health check-ups, pre-school education, weight measurements, and nutrition counseling) and the Public Distribution System (household has a Below Poverty Line card to obtain subsidized food). All models cluster standard error estimates at the state level. Sources: NFHS 4 (2016) for outcome and covariates. NSS-CES 50 (1994), 55 (2000) and 61 (2005) for MDM coverage data. MDM, mid-day meal; SES, socioeconomic status; GJ, Gujarat; OD, Odisha; CT, Chhattisgarh. Source data are provided as a Source Data file.

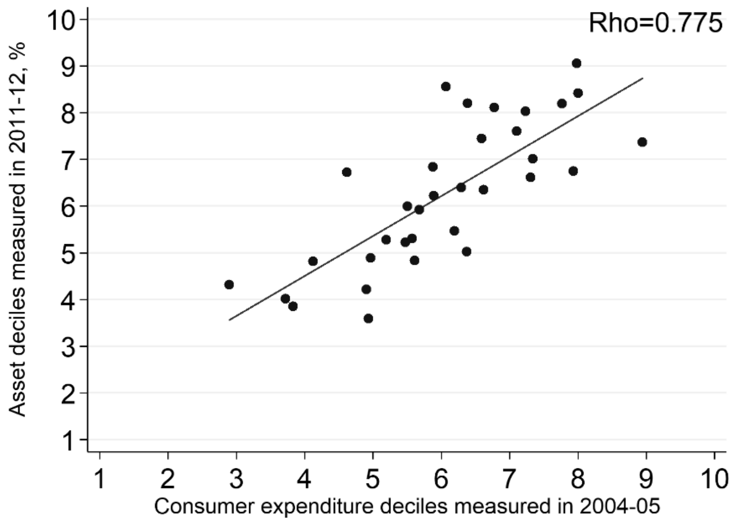

Supplementary Figure 3: State level correlation between deciles of monthly per-capita expenditure measured in 2005 and asset-based deciles in 2012.

Data source: India Human Development Survey (IHDS) rounds 1 (2005) and 2 (2012). Source data are provided as a Source Data file.

Supplementary Figure 4. Matching exposure to MDM by caste and religious groups instead of SES

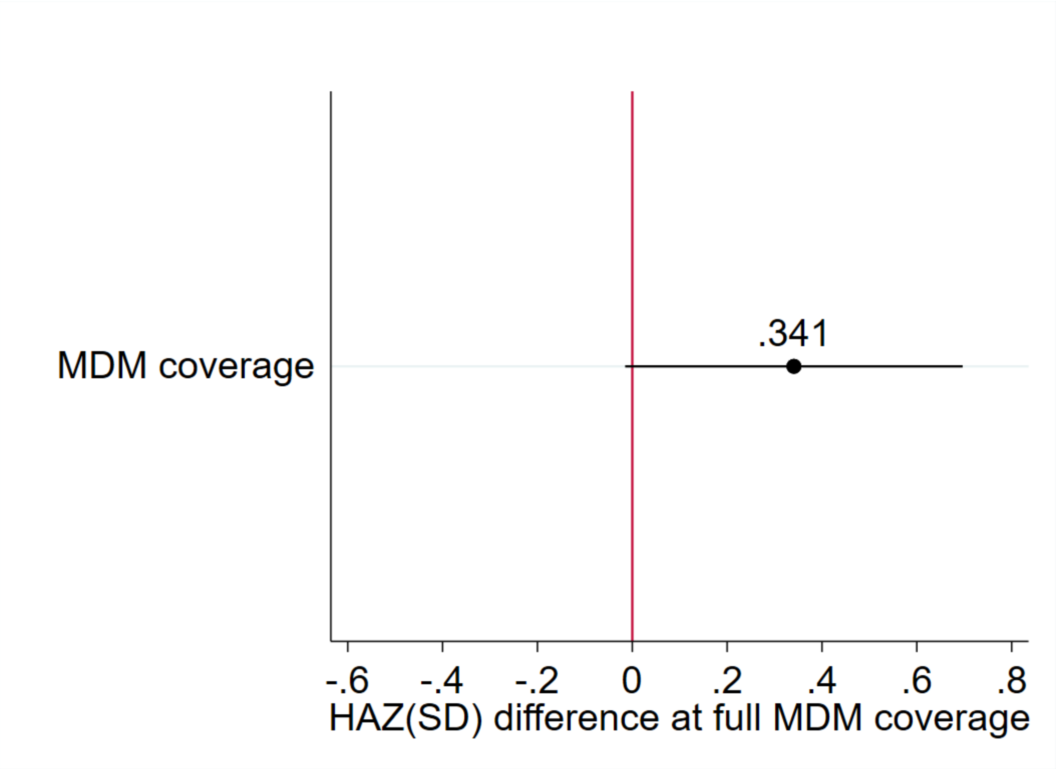

The circle represents the point estimates and whiskers are 95% confidence intervals. Point estimates are interpreted as the difference in HAZ due to 100% exposure to the MDM scheme during primary school years. MDM coverage is the proportion of girls born between 1980 and 1998, within state and sector (rural/urban) specific caste and religious group status deciles, who reported receiving at least 10 meals free of cost at school in the previous month. All models control for child age, sex, birth order, maternal antenatal care (4+ visits), institutional birth, residence (urban/rural), religion, caste, access to services from the Integrated Child Development Services (dummies for receiving take home rations, child health check-ups, pre-school education, weight measurements, and nutrition counseling) and the Public Distribution System (household has a Below Poverty Line card to obtain subsidized food). Sources: NFHS 4 (2016) for outcome and covariates. NSS-CES 50 (1994), 55 (2000) and 61 (2005) for MDM coverage data. MDM, mid-day meal; SES, socioeconomic status. Source data are provided as a Source Data file.

Supplementary Figure 5. Heterogeneity in program benefits among children who differ by height

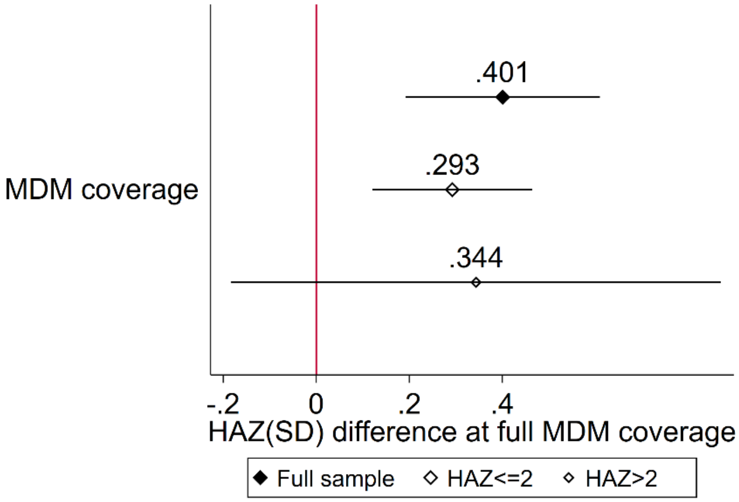

The diamonds represent the point estimates and whiskers are 95% confidence intervals. Point estimates are interpreted as the difference in HAZ due to 100% exposure to the MDM scheme during primary school years. MDM coverage is the proportion of girls born between 1980 and 1998, within state-specific caste and religious group status deciles, who reported receiving at least 10 meals free of cost at school in the previous month. All models control for child age, sex, birth order, maternal antenatal care (4+ visits), institutional birth, residence (urban/rural), religion, caste, access to services from the Integrated Child Development Services (dummies for receiving take home rations, child health check-ups, pre-school education, weight measurements, and nutrition counseling) and the Public Distribution System (household has a Below Poverty Line card to obtain subsidized food)**.** Sources: NFHS 4 (2016) for outcome and covariates. NSS-CES 50 (1994), 55 (2000) and 61 (2005) for MDM coverage data. MDM, mid-day meal; SES, socioeconomic status, HAZ=height for age z-score. Source data are provided as a Source Data file.

Supplementary Figure 6. Correspondence between raw MDM coverage data and interpolated smoothed coverage data

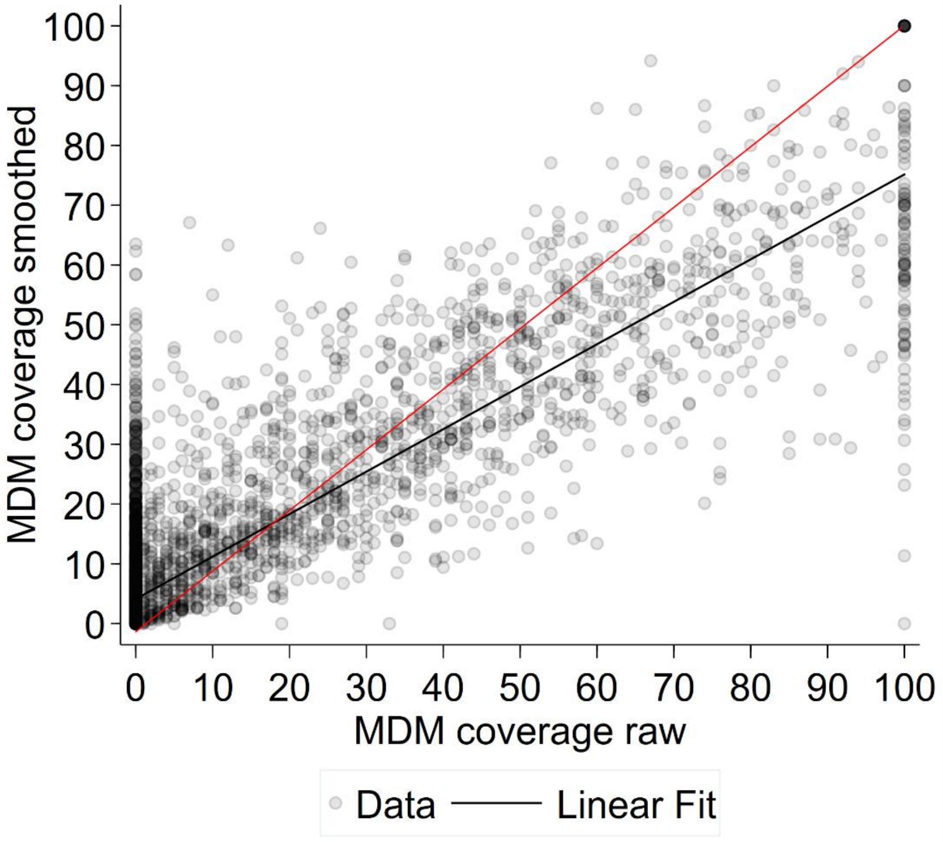

Circles represent data points and black line represent the linear fit of the data. The red line is the line of equality. The figure shows that the smoothed interpolated data are less extreme than the raw data. On average, smoothed interpolated values are smaller than raw data values. Source data are provided as a Source Data file.
