# Supplementary material for: Intergenerational nutrition benefits of India’s national school feeding program
Source: Nat Commun. 2021 Jul 12;12:4248. doi: 10.1038/s41467-021-24433-w (PMC8275765; doi:10.1038/s41467-021-24433-w)
Supplement: Supplementary file 3 — Reporting Summary [file 41467_2021_24433_MOESM3_ESM.pdf]

nature research

Corresponding author(s): Samuel Scott
Last updated by author(s): Mar 31, 2021

Reporting Summary

Nature Research wishes to improve the reproducibility of the work that we publish. This form provides structure for consistency and transparency in reporting. For further information on Nature Research policies, see our Editorial Policies and the Editorial Policy Checklist.

Statistics

For all statistical analyses, confirm that the following items are present in the figure legend, table legend, main text, or Methods section.

n/a	Confirmed	
☐	☒	The exact sample size (n) for each experimental group/condition, given as a discrete number and unit of measurement
☐	☒	A statement on whether measurements were taken from distinct samples or whether the same sample was measured repeatedly
☐	☒	The statistical test(s) used AND whether they are one- or two-sided<br>*Only common tests should be described solely by name; describe more complex techniques in the Methods section.*
☐	☒	A description of all covariates tested
☐	☒	A description of any assumptions or corrections, such as tests of normality and adjustment for multiple comparisons
☐	☒	A full description of the statistical parameters including central tendency (e.g. means) or other basic estimates (e.g. regression coefficient) AND variation (e.g. standard deviation) or associated estimates of uncertainty (e.g. confidence intervals)
☐	☒	For null hypothesis testing, the test statistic (e.g. F, t, r) with confidence intervals, effect sizes, degrees of freedom and P value noted<br>*Give P values as exact values whenever suitable.*
☒	☐	For Bayesian analysis, information on the choice of priors and Markov chain Monte Carlo settings
☒	☐	For hierarchical and complex designs, identification of the appropriate level for tests and full reporting of outcomes
☐	☒	Estimates of effect sizes (e.g. Cohen's d, Pearson's r), indicating how they were calculated

Our web collection on statistics for biologists contains articles on many of the points above.

Software and code

Policy information about availability of computer code

Data collection	No primary data were collected for this study. All data were drawn from publicly available sources including the Demographic Health Survey (National Family Health Survey (NFHS) wave 4), the National Sample Survey (NSS) of Household Consumer Expenditure, rounds 50, 55, 61 and 68, and the Indian Human Development Survey (IHDS) rounds 1 and 2. The NFHS data were collected by the International Institute for Population Sciences (IIPS) using computer assisted personal interviewing (CAPI) software and are available for download in STATA (.dta) format. The NSS data are collected by the National Sample Survey Organization (NSSO) of India. NSSO does not report the use of data collection software in technical reports. The final NSS data are available in a flat line-sequential ASCII format and need to be imported into STATA. The IHDS data were collected by India's National Council of Applied Economic Research (NCAER) using CAPI and are available for download in STATA format.
Data analysis	All analysis was performed using Stata software version 15. The code for the models used to generate the figures and tables in this manuscript will be made available in the IFPRI Dataverse, a member of the Harvard Dataverse (https://dataverse.harvard.edu/).

For manuscripts utilizing custom algorithms or software that are central to the research but not yet described in published literature, software must be made available to editors and reviewers. We strongly encourage code deposition in a community repository (e.g. GitHub). See the Nature Research guidelines for submitting code & software for further information.

Data

Policy information about availability of data

All manuscripts must include a data availability statement. This statement should provide the following information, where applicable:

- Accession codes, unique identifiers, or web links for publicly available datasets
- A list of figures that have associated raw data
- A description of any restrictions on data availability

All data that support the findings of this study are publicly available. National Sample Surveys of Consumer Expenditure data are available from the National Data Archive of the Ministry of Statistics & Programme Implementation [http://microdata.gov.in/nada43/index.php/catalog/CEXP]. National Family Health Survey data are available from IPUMS-DHS [https://www.idhsdata.org/idhs/]. The India Human Development Survey data are available from the IHDS website [https://www.ihds.umd.edu/data-download].

Field-specific reporting

Please select the one below that is the best fit for your research. If you are not sure, read the appropriate sections before making your selection.

☐ Life sciences ☒ Behavioural & social sciences ☐ Ecological, evolutionary & environmental sciences

For a reference copy of the document with all sections, see nature.com/documents/nr-reporting-summary-flat.pdf

Behavioural & social sciences study design

All studies must disclose on these points even when the disclosure is negative.

Study description

This study was a quasi-experimental secondary analysis of existing publicly-available quantitative observational data.

Research sample

NFHS4 (2016) is a national, state and district representative sample of 601,509 households with a response rate of 97.6%. In all households, women age 15-49 are eligible to participate. The study focuses on a sample of over 200,000 women with a mean age 26.8 years who had a living child in the 5 years preceding the survey (N=196386, 48% female, 52% male). The rationale for studying women and their children was based on prior evidence showing robust associations between mother's height and education and child growth. The exposure variable was obtained from the NSS-CES rounds 50 (1993/94), 55 (1999/00), 61 (2004/05), and 69 (2011/12). The NSS-CES cross sectional surveys are representative at the national and state levels. The NS-CES survey over 100,000 households in each round and provide information of demographic characteristics of all household members. The study used data on free meals obtained at schools, among girls aged 6-10 years, to calculate the exposure to the Mid-day Meal (MDM) program by state, socio-economic status (SES) and birth-year. The rationale for using data from girls was to match childhood exposure to free school meals to mothers in the NFHS using state, SES and birth-year as matching variables. The IHDS is a panel survey 42,152 households. It provided data on school meals and education and height of direct beneficiaries. The IHDS was used to verify assumptions in the primary models.

For a description of data sources, please see the 'Data sources' section in the manuscript and the Data Availability Statement.

Sampling strategy

NFHS4 follows at two-stage sample design selected with probability proportional to size followed by random sampling, separately for urban and rural areas. Since the NFHS4 has a large sample size and provides data by state, SES and birth-year of mothers, it was well suited to study intergenerational associations and obtain representative estimates. It covers mothers who were born before and during MDM implementation across states in India.

NSS follows a stratified multi-stage sampling design selected with probability proportional to size followed by random sampling, separately for urban and rural areas. We used 4 waves of the NSS-CES that provided data on school meals, spanning almost 20 years, to estimate state -SES -birth year specific changes in MDM coverage for mothers born between 1980 and 1998. We then matched these estimates to women in the NFHS4 sample. These birth years are sufficient for the analyses because they cover a sufficient proportion of women before and after program implementation to detect population level effects of the program.

The IHDS follows a multi-stage sampling design with a sampling frame selected from villages and urban blocks in India and representative at the national level. The IHDS data are only available for 2 waves, 7 years apart. These were ideal to investigate direct beneficiary relationships and test assumptions of the primary model. Being nationally representative, they were appropriate to make inferences at the country level.

Full details on sampling strategies are available from the dataset documentation accessible using the hyperlinks provided in the Data Availability Statement.

Data collection

For the main NFHS4 data, survey questionnaires were canvassed in 17 local languages using CAPI. The Household Questionnaire listed all usual members of the household and visitors who stayed in the household the night before the interview. Basic demographic information was collected on the characteristics of each person listed, such as age, sex, marital status, schooling, and relationship to the head of the household. The Woman's Questionnaire collected information from all eligible women age 15-49, who were asked questions on a large variety of topics, including reproduction, hygiene, family planning, contacts with community health workers, maternal and child health, marriage and sexual activity, fertility preferences, among others. The Biomarker Questionnaire covered measurements of height and weight for children and women. Data related to the biomarkers were initially recorded on the Biomarker Questionnaire and subsequently entered into the interviewers' mini-computers. The Seca 874 digital scale was used to measure the weight of children and adults. The height of adults and children age 24-59 months was measured with the Seca 213 stadiometer. The Seca 417 infantometer was used to measure the recumbent length of children under two years or less than 85 cm.

The pretest training was held in Kharghar, Navi Mumbai, from 25 November to 7 December 2013, and the pretest fieldwork was conducted from 8-12 December 2013. In all, 37 interviewers and 11 health investigators participated. Final data collection was

	conducted by 789 field teams. Each team consisted of one field supervisor, three female interviewers, one male interviewer, two health investigators, and a driver. The number of interviewing teams in each state varied according to the sample size. In each state, interviewers were hired by the selected Field Agencies, taking into consideration their educational background, experience, and other relevant qualifications. Female and male interviewers were assigned to interview respondents of the same sex. The field supervisor was responsible for the overall management of the field teams. In addition, the field supervisor conducted spot-checks to verify the accuracy of key information, particularly with respect to the eligibility of respondents. IIPS also appointed one or more project officers or senior project officers in each state for monitoring and supervision throughout the training and fieldwork period to ensure that correct survey procedures were followed and that data quality was maintained.<br><br>There was no experimental condition; all data were observational.<br><br>The protocol for the NFHS-4 survey, including the content of all the survey questionnaires, was approved by the IIPS Institutional Review Board and the ICF Institutional Review Board. The protocol was also reviewed by the U.S. Centers for Disease Control and Prevention (CDC).
Timing	Data collection for NFHS4 was conducted in two phases (from 20 January 2015 to 4 December 2016)
Data exclusions	From a sample of 259,627 women age 15-49 years who had children aged 0-5 years, those born between 1980 and 1998 (N=219,334) were retained in the final analytical sample.
Non-participation	During NFHS4 data collection, out of an eligible 723,875 women, N=699,686 were interviewed with a response rate of 96.7%. Of these 259,627 had children aged 0-5 years.
Randomization	Participants were not allocated into experimental groups. A continuous indicator coded as the proportion of mothers covered by the MDM as children and ranged between 0 and 1. Birth-year fixed effects which forces identification of within birth-year effects and controls for time-varying national level economic changes, programs and policies was included in the main model along with wealth-decile fixed effects which provides controls for all unobserved time invariant factors associated with household wealth and MDM coverage. State fixed effects were also included to control for all for time-invariant differences across states with high and low MDM exposure. An array of individual, household and survey specific controls was included: child age, sex, birth order, mothers antenatal care status during pregnancy, birth in a medical facility and household characteristics at the time the outcome was measured. The array also includes SES, caste, religion, and residence (urban or rural). Additionally, an array of individual and household specific programmatic controls including access to services from the Integrated Child Development Services (dummies for receiving take home rations, child health check-ups, pre-school education, weight measurements, and nutrition counseling) and the Public Distribution System (household has a Below Poverty Line card to obtain subsidized food) was included. Controlling for these variables reduces possible confounding from government interventions that could benefit current child nutritional status.

Reporting for specific materials, systems and methods

We require information from authors about some types of materials, experimental systems and methods used in many studies. Here, indicate whether each material, system or method listed is relevant to your study. If you are not sure if a list item applies to your research, read the appropriate section before selecting a response.

Materials & experimental systems

n/a	Involved in the study
☒	☐ Antibodies
☒	☐ Eukaryotic cell lines
☒	☐ Palaeontology and archaeology
☒	☐ Animals and other organisms
☒	☐ Human research participants
☒	☐ Clinical data
☒	☐ Dual use research of concern

Methods

n/a	Involved in the study
☒	☐ ChIP-seq
☒	☐ Flow cytometry
☒	☐ MRI-based neuroimaging
