# Supplementary material for: Intergenerational nutrition benefits of India’s national school feeding program
Source: Nat Commun. 2021 Jul 12;12:4248. doi: 10.1038/s41467-021-24433-w (PMC8275765; doi:10.1038/s41467-021-24433-w)
Supplement: Supplementary file 2 — Peer Review File [file 41467_2021_24433_MOESM2_ESM.pdf]

Editorial Note: This manuscript has been previously reviewed at another journal that is not operating a transparent peer review scheme. This document only contains reviewer comments and rebuttal letters for versions considered at *Nature Communications*. Mentions of prior referee reports have been redacted.
